# Preclinical Models for Alzheimer’s Disease: Past, Present, and Future Approaches

**DOI:** 10.1021/acsomega.2c05609

**Published:** 2022-12-13

**Authors:** Ansab Akhtar, Shraddha M. Gupta, Shubham Dwivedi, Devendra Kumar, Mohd. Farooq Shaikh, Arvind Negi

**Affiliations:** † Department of Pharmaceutical Sciences, School of Health Sciences and Technology, UPES, Dehradun, Uttarakhand, Dehradun 248007, India; ‡ Faculty of Pharmacy, DIT University, Uttarakhand, Dehradun 248009, India; § Neuropharmacology Research Strength, Jeffrey Cheah School of Medicine and Health Sciences, Monash University Malaysia, Bandar Sunway, Selangor 47500, Malaysia; ∥ Department of Bioproducts and Biosystems, Aalto University, FI-00076 Espoo, Finland

## Abstract

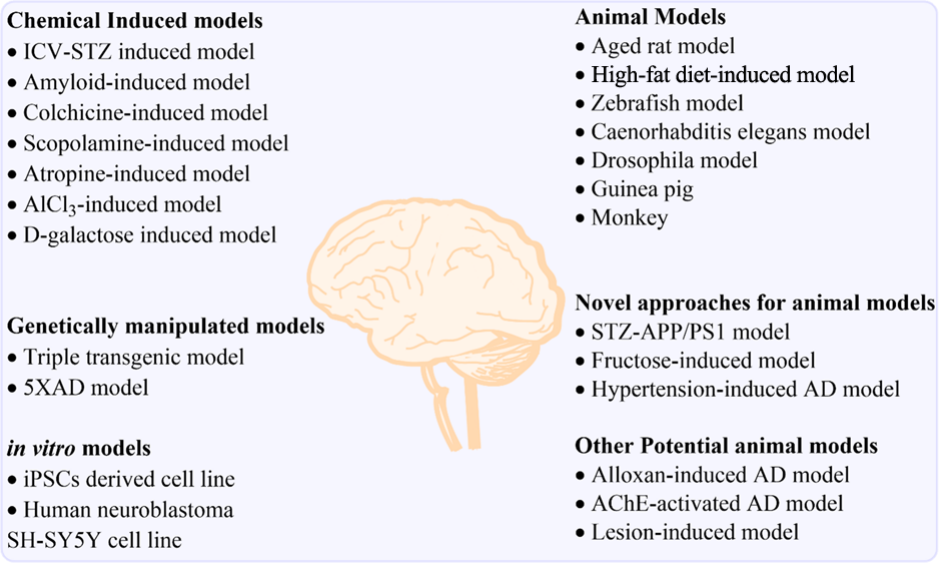

A robust preclinical disease model is a primary requirement to understand the underlying mechanisms, signaling pathways, and drug screening for human diseases. Although various preclinical models are available for several diseases, clinical models for Alzheimer’s disease (AD) remain underdeveloped and inaccurate. The pathophysiology of AD mainly includes the presence of amyloid plaques and neurofibrillary tangles (NFT). Furthermore, neuroinflammation and free radical generation also contribute to AD. Currently, there is a wide gap in scientific approaches to preventing AD progression. Most of the available drugs are limited to symptomatic relief and improve deteriorating cognitive functions. To mimic the pathogenesis of human AD, animal models like 3XTg-AD and 5XFAD are the primarily used mice models in AD therapeutics. Animal models for AD include intracerebroventricular-streptozotocin (ICV-STZ), amyloid beta-induced, colchicine-induced, etc., focusing on parameters such as cognitive decline and dementia. Unfortunately, the translational rate of the potential drug candidates in clinical trials is poor due to limitations in imitating human AD pathology in animal models. Therefore, the available preclinical models possess a gap in AD modeling. This paper presents an outline that critically assesses the applicability and limitations of the current approaches in disease modeling for AD. Also, we attempted to provide key suggestions for the best-fit model to evaluate potential therapies, which might improve therapy translation from preclinical studies to patients with AD.

## 1 Introduction

Alzheimer’s is a progressive neurodegenerative disease characterized by the deposition of amyloid plaques and neurofibrillary tangles (NFT) in the brain regions of the cerebral cortex and hippocampus, which are the major pathological hallmarks of the disease. The common symptoms of the disease include memory loss, impairment in learning, and retarded intellectual and thinking ability. However, the number of patients with AD-related dementia is expected to reach 152 million globally by the year 2050, with the highest growth anticipated in low-to-middle-income nations.^1^ According to 2020 statistics, AD patients (≤65 years) in the United States may increase dramatically from 5.8 million to 13.8 million by 2050.^2^ Further population studies conducted in Japan and China showed a marked increase in AD prevalence in the past several years.^3^

**Figure 1:**
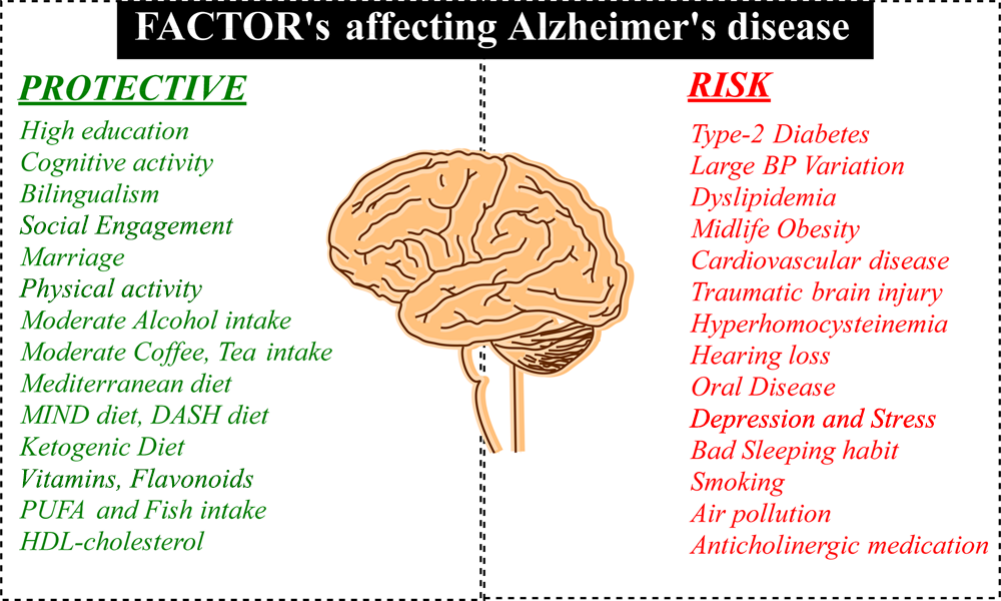
Potential risk factors for AD (PUFA = polyunsaturated fatty acid; HDL-cholesterol = high-density lipoprotein cholesterol).^6^

Additionally, various risk factors contribute to AD progression and symptoms, as shown in Figure 1. Therefore, a proper diagnosis is crucial for patients with cognitive impairment. Significantly, amyloid precursor protein (APP)-mediated generation of amyloid-β (Aβ) and hyperphosphorylated tau protein-generated neurofibrillary tangles are the major pathological hallmarks of AD. Hence, Aβ and tau are the indicative biomarkers of AD. Still, healthy individuals with these biomarkers at normal or above-normal levels often do not show signs and symptoms of AD,^4^ making it challenging to get a presymptomatic diagnosis. Future obstacles would include the discovery of less invasive and more sensitive biomarkers or procedures that can also be employed for early detection and diagnosis. In any case, future research must investigate evidence-based preventative techniques consistent with the apparent relationship between modifiable risk variables and late-onset AD.

According to data from prospective population-based research, the majority of risk variables were pre-existing diseases, poor lifestyles, and environmental exposures that significantly impact the likelihood of dementia in old age.^5^ Still, psychological circumstances and healthy lifestyles may protect against AD. In addition, many elements appeared to represent both AD risk factors and symptoms, presumably due to reverse causality; these factors are emphasized in Figure 1.^6^ Late-onset AD is a complex genetic disorder with a 60–80% hereditary rate. APOE genotype is the most significant risk factor for late-onset AD.^7^

**Figure 2:**
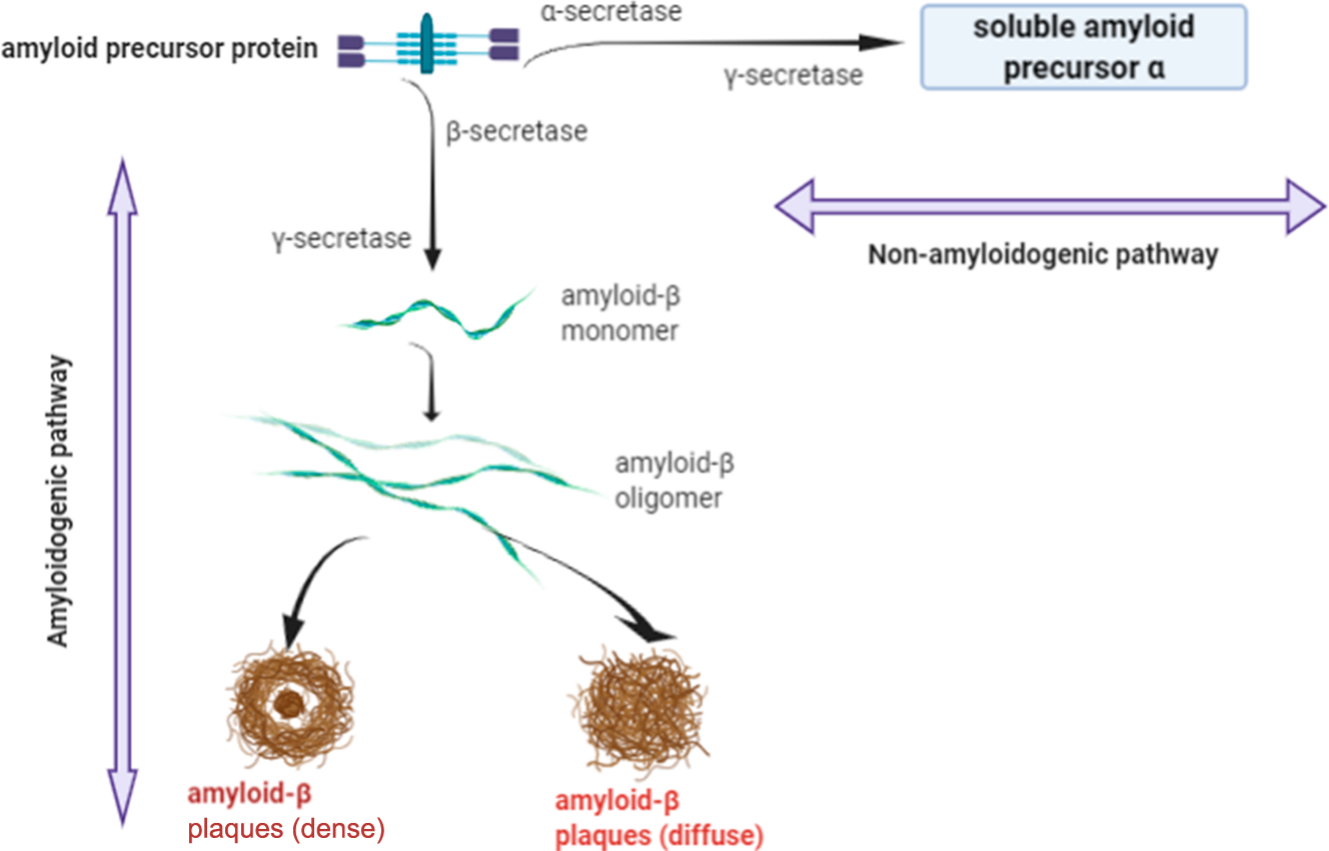
Amyloidogenic and nonamyloidogenic pathways.

Extracellular amyloid plaques formed by amyloid precursor protein (APP) and intracellular neurofibrillary tangles resulting from hyperphosphorylated tau protein in the cortical and hippocampal regions are the key pathological hallmarks of AD.^8^ The catalytic proteases involved in APP proteolysis include α-, β-, and γ-secretases. This process forms the basis of the amyloidogenic pathway with β- and γ-secretase and the nonamyloidogenic pathway with α-secretase, generating insoluble and soluble neurotic amyloid plaques, respectively (Figure 2).^9^

**Figure 3:**
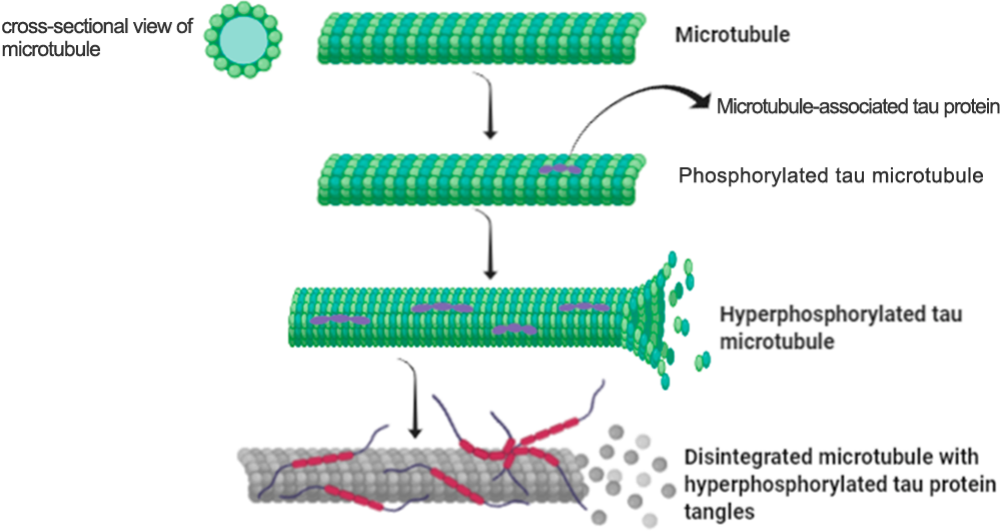
Hyperphosphorylated tau protein-mediated tangles.

Tau contains 441 amino acids, where 85 potential amino acids (serine, threonine, and tyrosine) are available for phosphorylation. Typically, two to three locations of tau are reported to be phosphorylated in normal physiological conditions. However, tau hyperphosphorylation is a pathological condition having phosphorylation at almost six positions (as shown in Figure 3).^10^

**Figure 4:**
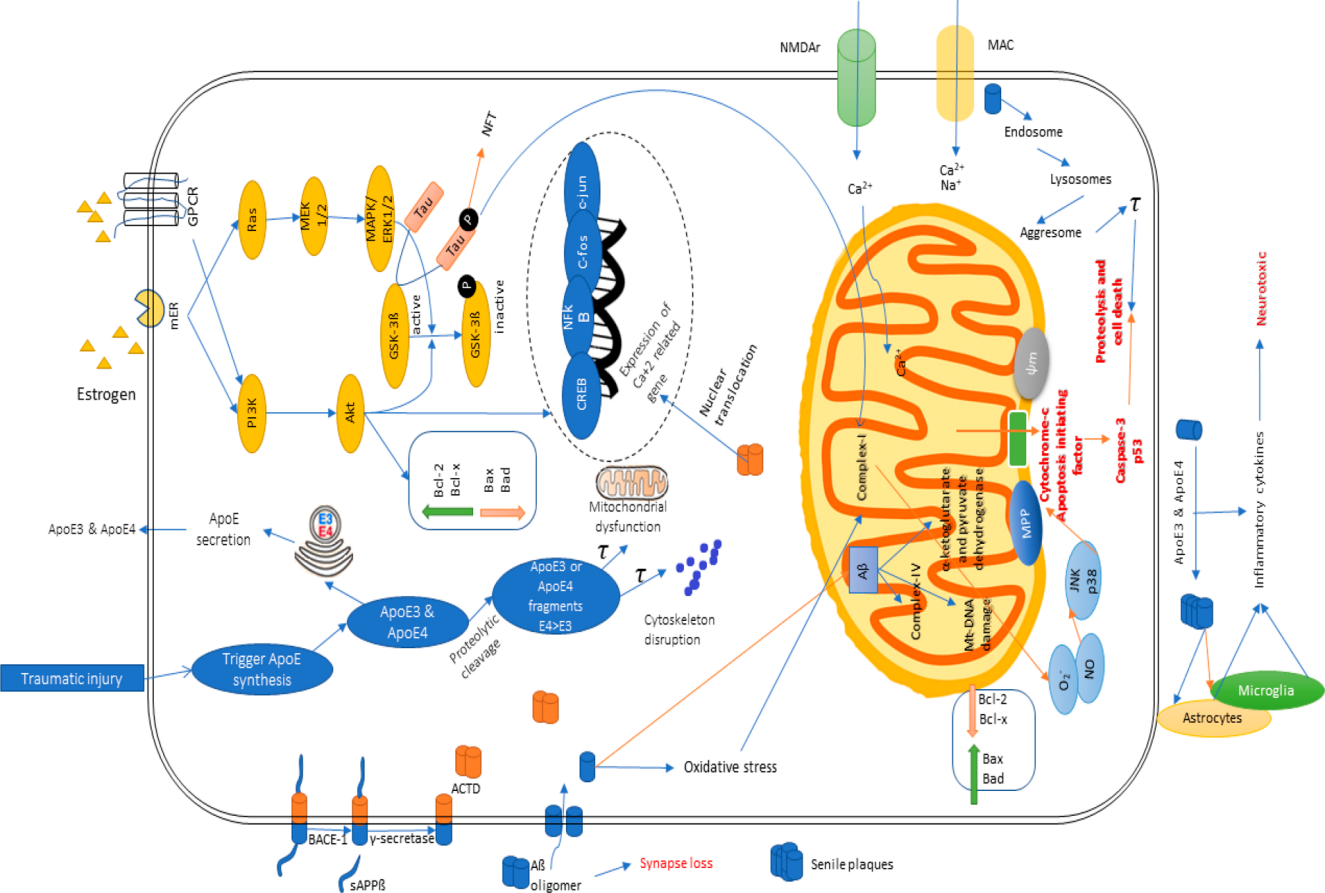
Various pathophysiological pathways of Alzheimer’s disease.

Notably, AD has been demonstrated to depict underlying mechanisms through numerous cascades, including reactive oxidative species or oxidative stress, mitochondrial dysfunction, cholinergic dysfunction, neuro-inflammation, glucose metabolism impairment, and, most importantly, insulin signaling pathway dysregulation or insulin resistance^11^ (as shown in Figure 4). Therefore, targeting these pathways through an inhibitory way can be advantageous and beneficial to minimize AD-related deterioration and symptoms and also retard the neurodegenerative process and disease progression in an advanced case. Animal models to carry over these harmful mechanisms (oxidative stress, mitochondrial dysfunction, cholinergic dysfunction, neuroinflammation, and insulin resistance) will be suitable for finding newer therapeutic interventions and pharmacological treatments against AD.

The currently approved drugs are either acetylcholinesterase inhibitors (such as rivastigmine, donepezil, and galantamine) or NMDA receptor antagonists (like memantine). However, these drugs only have limited symptomatic relief but fail to prevent AD progression. Several newer molecules that have been identified for AD in the last four decades act by preventing amyloid deposition in the brain and removing existing amyloid plaques along with other recognized mechanisms associated with the disease.^12^ Although these compounds demonstrate promising data from preclinical AD models, the clinical failure rate in AD treatment is almost 100%. Several factors can be alleged for the failure, but the primary concern remains with the preclinical models used at the preliminary stages of drug development. This indicates the gap between the preclinical data curation and their translational value. The flawed and incomplete preclinical evaluation of candidate drugs for AD may be the cause of the molecules’ blip into clinical trials. Hence, in the context of the significant shortcomings of preclinical models, the reasons for poor translation to clinical outcome could be poor hypothesis or target selection, model selection, validation, and pharmacodynamic/pharmacokinetic (PD/PK) characterization of novel drugs.

Moreover, on the basis of amyloid theory and genetics of AD, a rising dependency on transgenic AD models possessing the targets of amyloid plaque and tau protein has been witnessed.^13^ Additionally, the mouse brain produces amyloid peptides distinct from the human brain, and the mouse model, even with amyloid deposition, often fails to show a substantial neuronal loss.^14^ Moreover, comorbidities associated with human AD are not well-mimicked in animal models. Despite numerous limitations associated with the preclinical AD model, these models can somewhat predict the pathophysiology and therapeutic targets. However, selecting a suitable model system for the specific target/pathway with scientific rationalization can reduce the chances of failure in drug screening. This review aims to provide insight into the rationalization and selection of animal models for AD. The objective is to discuss the current and future animal models for AD and their role in exploring pathophysiology/drug development. Despite decent face validity, transgenic models usually lack complete content and predictive validity. In this review, we have discussed the current preclinical AD models used in drug screening for AD. This review should be helpful in guiding researchers toward the rational selection of preclinical models for AD.

## 2 Chemically Induced Central Administration

### 2.1 ICV-STZ-Induced Model

Sporadic Alzheimer’s disease (SAD) is a multifactorial disease caused by genetic, epigenetic, environmental, and metabolic factors. Among metabolic factors, impaired glucose metabolism and energy utilization are observed in the initial stages of disease progression.^15^ In this regard, the animal models have made considerable progress in unveiling the molecular pathways involved in AD’s pathogenesis; thus, it has led to the development of potential therapeutic approaches for AD. One such animal model that is widely used is to produce insulin signaling impairment through intracerebroventricular (ICV) streptozotocin (STZ) administration.

There is an abundance of insulin receptors in brain regions like the cortex, hippocampus, hypothalamus, olfactory bulb, etc.; clinical reports showed downregulation of insulin, insulin receptors, and insulin receptor substrates under the influence of ICV-STZ-mediated insulin resistance.^11,16−18^ Other AD phenomena associated with the ICV-STZ model include oxidative stress, mitochondrial dysfunction, cholinergic dysfunction, and neuroinflammation. These factors are considered to be the triggering points of neurodegeneration and are adjacently interlinked with insulin resistance.^11,16−18^ Hence, these characteristics associated with the neuropathology of the ICV-STZ model have the potential to lead to cognitive deficit and memory impairment, signifying a strong reason to validate the sporadic AD model. Various studies have been carried out in the past and recently to confirm and validate the ICV-STZ-induced SAD model and its potential to generate brain insulin resistance, as seen in SAD patients.^11,16,17^ Hence, it aims to provide insights into the molecular mechanisms involved in causing brain IR-induced AD by ICV-STZ, and further, its advantages and limitations are elaborated. Recent reports of the ICV-STZ model have depicted the downregulation of the α7-nicotinic acetylcholine receptor (α7AchR). This report has shown it to attenuate disease pathology with the insulin-sensitizing agent metformin signifying the role of the cholinergic and insulin signaling pathway in the ICV-STZ model of AD.^19^

### 2.2 Amyloid-Induced Model

As a replacement to ICV-STZ, the amyloid-β-42, amyloid-β-40, and amyloid-β-25–30 can be administered via the intracerebroventricular or intrahippocampal route. These amyloids have been subcategorized based on the number of amino acids possessed by them. Moreover, they have different degrees of pathogenesis in AD, where amyloid-β-42 is the most pathogenic one. The ICV-Aβ injection has been proved to drive the neurodegenerative process and impairment in learning and memory. This outcome could happen through normalizing oxidative and nitrosative parameters.^20^ There has been an excessive generation of reactive oxygen species (ROS) due to amyloid-β.^21^ Moreover, APP processing is enhanced in an Aβ-42 model of AD, creating more deposits of senile plaques.^22^ There is a downregulation of nicotinic acetylcholine receptors in an amyloid-induced model. This downregulation leads to cholinergic dysfunction.^23^ Amyloid also has a role in transgenic mouse models.^24^ Further, the central administration of Aβ also leads to the formation of tau-related tangles. Mitochondrial dysfunction through oxidative stress pathways can also arise.^25^ Amyloid is one of the core pathological hallmarks of AD, has been made to be targeted through a monoclonal antibody aducanumab in the latest development for the therapy of AD, and, hence, has been granted accelerated approval by the U.S. Food and Drug Administration (FDA). This approval is significant versus other conventional treatments of AD as this antibody acts as an antiamyloid-β factor and thereby can halt the progression of the disease pathology and neurodegeneration.^26^

## 3 Chemically Induced Oral Administration

### 3.1 Colchicine-Induced Model

Colchicine has been clinically used for gout treatment and is naturally obtained from a plant species (*Colchicum autumnale*). It has a unique capacity to bind to microtubule-associated tubulin protein. It causes the destabilization of microtubules and is generally administered through the oral route for treatment.^27^ However, a dose of 15 μg in a 5 μL vehicle, like distilled water in rats, produces cognitive impairment. The cognitive impairment produced is quite comparable to sporadic AD upon intracerebroventricular administration. Colchicine destroys oxidative balance and cholinergic pathways and aggravates the neuroinflammatory pathways responsible for synaptic dysfunction and neurodegeneration.^28^ Cycloxygenase-2 (COX-2), prostaglandinE2 (PGE2), interleukin-β (IL-1β), and tumor necrosis factor-α (TNF-α) might be responsible for inflammatory action in the colchicine-induced model. Furthermore, microtubules, the central building part of the axonal and neuronal cytoskeleton, cause significant deterioration, thereby paving the way to neuronal death.^29^

## 4 Chemically Induced Intraperitoneal Administration

### 4.1 Scopolamine-Induced Model

Scopolamine is a tropane alkaloid, also known as hyoscine. It is a potent anticholinergic drug obtained from *Hyoscyamus niger*. It is generally used before traveling to prevent motion sickness and after surgery to check nausea and vomiting. It is a competitive inhibitor of muscarinic receptors and is useful in many cholinergic-related discomforts and side effects such as increased bowel movements, salivation, lacrimation, sweating, etc.^30^ Acetylcholine is one of the most crucial neurotransmitters in memory processing by strengthening synaptic connections; hence, scopolamine-mediated blockage of cholinergic nervation is widely used as an animal model of AD. Scopolamine acts by the increased acetylcholinesterase (AchE) activity, enhancing the breakdown of acetylcholine. The dose of scopolamine is ∼2 mg/kg intraperitoneally for an AD model. In this process, scopolamine disrupts several brain regions’ connectivities like spatial memory mapping and functional network.^31^ The advantage of the scopolamine-induced model is the avoidance of complex surgical procedures like in an ICV model. Furthermore, cholinergic drugs like donepezil and rivastigmine and antioxidants like melatonin have been demonstrated to reverse scopolamine-induced memory impairment, proving the additional involvement of the oxidative stress pathway.^32,33^ Therefore, this model is mainly preferred for creating preventive options in AD treatment.^34^

### 4.2 Atropine-Induced Model

Atropine is also an alkaloidal origin drug obtained from *Atropa belladonna* and has been used as an anticholinergic drug to treat low heart rate and myopia. Atropine, similar to scopolamine, invades the cholinergic pathway, reducing the muscarinic Ach receptor’s hypofunction. It also blocks the nicotinic one up to a minor extent.^35,36^ Atropine in a dose of 5 mg/kg intraperitoneally (ip) for 21 days generated amyloid plaques, a pathological hallmark of AD. This process could result from an interlink between the cholinergic pathway and amyloidogenesis.^35,37^ Furthermore, the reduced release of acetylcholine inflicted by Aβ and vice versa was observed.^38^

### 4.3 Aluminum Chloride-Induced Model

Aluminum is an element that in excess causes numerous toxicities. An AD model can be established in rats or mice by ip injection of 4 mg/kg or 40 mL/kg per day of aluminum chloride (AlCl_3_) for nearly 40 days.^39^ The weighty triggers in an AlCl_3_ model are oxidative stress and mitochondrial dysfunction, which are reported to appear by inhibiting the NADH dehydrogenase enzyme of the electron transport chain.^40^ These phenomena were precisely reported in memory centers of the cortex and hippocampus. Further, neuroinflammatory mediators, including iNOS, NF-κB, COX-2, and proinflammatory cytokines, are altered in an AlCl_3_ model, leading to neurodegeneration. Additionally, any aluminum salt in doses of 100 mg in 1 day or 20 mg in 5 days has also been found to induce AD-associated neurotoxicity.^41^ Further, Al salts also cause cholinergic dysfunction and oxidative stress, leading to the apoptotic process.^42^ This model has also been preferred for prophylaxis treatment of AD. In this way, the therapeutic agents can be made available as a preventive measure rather than a protective one by utilizing this model.^43^

## 5 Chemically Induced Subcutaneous Administration

### 5.1 d-Galactose-Induced Model

d-Galactose is a monosaccharide in dairy products, avocados, sugar beets, etc. (e.g., milk contains 7.12 mg of galactose per 100 g; 100 g of avocado contains 0.66 g of sugar, which includes glucose, fructose, sucrose, and galactose; and sugar beet has 0.65% galactose). The metabolism of d-galactose produces ROS.^44^d-Galactose in doses of 50, 100, and 200 mg/kg through the subcutaneous route increased escape latency in the Morris water maze (MWM) and lowered the discrimination index in the novel object recognition (NOR) test in a dose-dependent way. This increment shows impaired spatial and recognition memory. Further, it increases oxidative stress in the hippocampus.^45,46^ There are reports of the attenuated immune system upon d-galactose treatment mimicking an aging brain.^45,47^ It also halts neurogenesis in the hippocampus and dentate gyrus regions of the brain^48^ and disrupts calcium homeostasis in the cortex and hippocampus, creating excitotoxicity conditions similar to those found in dementia cases.^49^ This model can be used in insulin resistance-associated AD cases because d-galactose is a sugar and produces an insulin-resistance-like state.

## 6 Genetically Manipulated Model

### 6.1 Triple Transgenic Model

The triple transgenic model is a model of an inherited familial form of AD involving mutations on three genes, such as APP on chromosome 21, presenilin 1 on chromosome 14, and p-tau in mice, hence named the triple transgenic model. The mutations in these genes might lead to AD pathogenesis because AAP and tau are linked to amyloid plaques and NFT, respectively, whereas presenilin 1 is the proteolytic subunit of γ-secretase (involved in APP cleavage). For the development of the model, transgenes encoding the mentioned ones are microinjected into mice. The mutations can happen by knocking in APP-Swe, PS1-M146 V, and tau-P301L. Crossing the mutant mice can also result in a familial AD (FAD) model.^50,51^ In addition, the development of both amyloid oligomers and paired helical filaments of tau was studied.^52^

Further, this transgenic model demonstrates brain atrophy, synaptic disruption, and neuronal death and cannot regenerate neurons in the areas of the prefrontal cortex, hippocampus, and dentate gyrus, leading to cognitive decline and memory impairment.^53^ Both spatial and recognition memory were found to be impaired in transgenic mice. Other than cognitive impairment, phenotypic alterations were also reported due to mutated mice.^54^ A transgenic mouse with mutations at APP and presenilin 2 on chromosome 1 or only at APP can also be created with closely related features to FAD.^55^ Even though the mouse models of APP^G-F^ for BACE1 inhibitors (BACE1 is beta-site APP-cleaving enzyme 1 responsible for breaking down APP) and APP^P11JL^ for immunotherapies have been discovered, these are not suitable for the more prevalent sporadic AD.^56^

### 6.2 5XAD Model

5XAD is another transgenic mouse model of FAD, denoting mutations in five genes. 5XAD expresses APP695 with S-K670N, S-M671L, F-I716 V, and L-V717I mutant genes.^57^ These genes are the types of APP mutations expressed in mice. The mutations in these genes lead to AD pathology. These mutations result in the excessive production of senile plaques from APP. Moreover, this model also represents gliosis, synaptic disruption, and neuronal death.^58^ The model depicts the features of AD earlier than in other transgenic models; however, phosphorylated tau pathology is less prevalent than amyloid plaques in this model.^52^ Further, proinflammatory cytokines and immune markers through microglial and caspase-3 activation in the brain regions of the cortex and hippocampus have been reported, indicating neuroinflammation and subsequent apoptosis-generated^59^ neurodegeneration.^60^

## 7 Animal Models without Chemical Induction or Genetic Modification

### 7.1 Aged Rat Model

Compared to younger ones, aged rats show damage triggered naturally in the hippocampus, temporal lobe, and neocortex, which subsequently causes impairment in learning and memory. This model is preferred over other chemical-induced models due to its noninvasive influence and mimicking late-onset/aged sporadic AD pathological symptoms. The age range of rats used for this model could be taken between 15 and 20 months old. This model is more relevant considering the disease’s clinical aspects.^61^ Further, aging-induced dementia has depicted neuroinflammatory cytokines, oxidative stress, insulin resistance, and mitochondrial dysfunction resulting from an old age-related phenomena like glucose and energy metabolism, obesity, physical inactivity, etc. This model also produces the condition of amyloidogenesis and tau pathology comparable to other models of AD. Exercise, intermittent fasting, and several other antiaging measures have been proven to reverse these detrimental features of AD, leading to improved synaptic plasticity and memory formation.^62^

### 7.2 High-Fat Diet-Induced Model

A high-fat diet is widely used to create a model for insulin resistance, obesity, and diabetes mellitus. However, in several recent research reports, it has also been recommended to be designated as a cognitive dysfunction model. In addition to the peripheral distortion of insulin sensitivity, providing fat-loaded diets for almost 10–14 weeks to the rats or mice instead of a regular diet also potentially induces central insulin resistance up to some extent.^63,64^ The fatty diet comprises 25% fat, 20% protein, and 50% carbohydrate.^64^ Dementia and AD have long been characterized to possess distorted brain insulin signaling. This model has a core feature of insulin resistance that is relevant to evaluating memory and improving therapeutic interventions.

Moreover, high-fat diet-induced obesity hampers proper blood flow to the brain regions, reducing oxygen and glucose supply and resulting in vascular dementia. Besides, hypertension and diabetes-induced cognitive decline has also been reflected in situations of a high amount of dietary fat intake.^65^ Fat-associated cholesterol has a role in the generation of senile plaques by upregulating APP, which is accountable for neuronal loss.^66^ The other mechanisms involved in memory loss could also be an imbalance in lipid profile and glucose-transport interference. The high-fat diet AD model also exacerbates oxidative stress and neuroinflammation through decreased antioxidant enzymes and increased proinflammatory cytokines.^67^

## 8 Animal Models Other than Rat/Mouse Species

### 8.1 Zebrafish Model

Zebrafish are a freshwater fish found in tropical areas. It has been considered a comfortable and conspicuous model for cellular, molecular, and genetic studies as they have a conspicuous molecular structure and the cellular network is not complex. Mutant genes of APP and presenilin orthologues have been discovered in zebrafish embryos, making it a relevant FAD model.^68^ This model has numerous advantages over rodent models, such as the optically transparent embryo structure. Also, fewer neurons form a clear picture of the neuronal network, rapid neurogenesis, neuronal development, significant reproductive behavior, and swift manipulations of genetic makeup.^69^ Smaller size, simpler tissue organization, and high fertility rate make it even more appropriate for high-throughput screening of novel drugs.^70^ However, limitations include a higher mortality rate, difficulty in maintenance, and lesser resemblance to human physiology than rodents. Apart from natural mutants in zebrafish, artificially created mutations have also been revealed.^71^ Several genome editing tools in zebrafish include zinc finger nuclease, transcription activator-like effector nuclease, CRISPR, etc. Both knock-out and knock-in methods can be used for genetic manipulations in zebrafish that are suitable for studies on neurodegenerative diseases, specifically AD.^72^

### 8.2 *Caenorhabditis elegans* Model

*C. elegans* belong to nematodes, and in the last few decades, it has been extensively used as a model for studying human diseases, specifically neurodegenerative diseases. Concerning its anatomy, it is transparent and quite less complicated as compared to rodents and humans. It has almost 40% ortholog genes of APP and tau and has a pivotal role in AD pathogenesis,^73^ making it appropriate for the revelations of AD genomic-level research. The model’s other advantages are its high breeding power, lower food requirement, and visible neurons under a microscope. However, the model’s disadvantages are a short life span and a smaller size to handle. This is a reliable model for evaluating spatial memory and exploratory behavior.^74^

Furthermore, the synapses of *C. elegans* are flexible to modify. Hence, agents that are potentially investigated to target synaptic functions and behavioral parameters find a space here and determine synaptic plasticity and memory processing.^75^ A transgenic *C. elegans* model has also been established for studying amyloidogenesis linked to FAD.^76^

### 8.3 Drosophila Model

The fruit fly *Drosophila melanogaster* is another model for cellular and molecular findings of neurodenerative diseases and, thus, is helpful as an AD model. This model better mimics symptoms of sporadic or late-onset AD. The model primarily involves the expression of APP, BACE-1, presenilin, and tau orthologs resulting in amyloid aggregation in the model brain, leading to neurodegeneration and memory loss.^77,78^ Here, the tau ortholog is the homologous tau genes of *Drosophila* with humans. Further, presenilin (proteolytic subunit of γ-secretase) regulates the cleavage of other proteins like APP, the mutation of which can generate AD pathology. Additionally, it is also applicable in several other neurodegenerative diseases’ biochemical studies due to its short life span and rapid generation ability.^79^ The neuronal network in Drosophila has made it a robust model for understanding memory acquisition and consolidation mechanisms. The orthologs found in the Drosophila genome are AAPl and dBACE, where APPl has nearly 30% similarity to human APP, providing profound insights into amyloid-related toxins’ research.^80^ Additionally, another amyloidogenic enzyme γ-secretase has also been detected in Drosophila. On a further note, genetic-based transgenic Drosophila has also been essential in FAD-related dementia, which overexpresses Aβ in the central nervous system (CNS).^78,81^

### 8.4 Guinea Pig

Unlike rats and mice, the guinea pig (*Cavia porcellus*), a nontransgenic animal model, has a human-like Aβ peptide sequence. High-cholesterol diets enhance BACE1 (β-secretase) transcription and decrease ADAM10 (α-secretase) transcription, which should increase Aβ release from APP.^82^

Guinea pigs have AD-related isoforms not observed in mice or rats. The guinea pig tau gene, MAPT, encodes isoforms with three and four microtubule-binding domains, like humans but unlike mice. Cholesterol affects the ratio of these isoforms. Guinea pigs are a good model for studying how dietary variables like cholesterol affect AD-related genes. Their AD-related genes are more human-like than rats or mice.^82^

Guinea pigs are the only small animal model where PS2V generation has been discovered. Human neuroblastoma cells exposed to hypoxia-induced oxidative stress and the brains of people with sporadic, late-onset Alzheimer’s disease had previously been shown to express the PS2V transcript.^83^

### 8.5 Monkey

Nonhuman primates have the potential to serve as valuable models of sporadic age-related brain-amyloid deposition as well as the pathologic alterations associated with AD. Some nonhuman primates can develop signs of AD during the aging process that are strikingly comparable to those of people with the disease (Table 1). These symptoms include neuropathy and changes in cognitive and behavioral patterns. Aging animals, on the other hand, are not models of Alzheimer’s disease; instead, they are good models of normal aging and naturally occurring Aβ deposition, and some display cognitive impairment. Deposition of amyloid in the brain parenchyma has been seen in the vast majority of nonhuman primates up to this point, which include rhesus monkeys,^84^ chimpanzees,^85^ vervet monkeys,^86^ marmosets,^87^ and cynomolgus monkeys.^88^

**Table 1:** Monkey Models of AD

animal species	rhesus macaques	stump-tailed macaques	mouse lemurs	common marmoset	cynomolgus monkeys
scientific name	*Macaca mulatta*	*Macaca arctoides*	*Microcebus murinus*	*Callithrix jacchus*	*Macaca fascicularis*
body length	45–64 cm	45–70 cm	12–13 cm	10–12 cm	40–65 cm
weight	5–12 kg	7–12 kg	50–100 g	80–100 g	9 g
lifespan	34–40 years	>30 years	14 years	7–11 years	35 years
age when considered old	20 years	24 years	5 years	7 years	20 years

## 9 In Vitro Models

In vitro models can also reproduce the disease model at cellular and molecular levels. However, the in vitro models’ robustness is not as profound as compared to in vivo models.

### 9.1 Neuroblastoma Cell Line

The neuroblastoma cell lines, also known as SH-SY5Y cell lines, can potentially develop neuronal cells functioning as neurons upon treatment with various agents. SH-SY5Y cell lines are obtained from neuroblastoma with the subcloning technique. Neuroblastoma is constituted of Schwann cells and neuroblasts. Therefore, these cell lines can be utilized to develop potential therapeutic agents for treating AD.^89^

### 9.2 iPSC-Derived Cell Lines

The generation of induced pluripotent stem cells (iPSC) from AD patients and differentiating them into neuronal cells has been regarded as a well-known model for AD-related studies. Conventional models do not recapitulate the complex form of SAD; hence, human-induced PSC can rejuvenate this field by filling the vacuum. This in vitro model can potentially create a brain-like microenvironment mimicking AD patients.^90^ The pathological hallmarks like amyloid plaques and NFTs centered at iPSC can be the basis of the model. This model can help study AD pathology and find potential therapeutic drugs for the disease. Familial and sporadic AD can be the model’s basis depending on the patient’s source.^91^

## 10 Novel Approaches for AD Animal Model

### 10.1 STZ-APP/PS1 Model

This STZ-APP/PS1 dual model is a newer approach to treating memory impairment in mice. The strategy combines previously used STZ-induced dementia and a genetically modified transgenic model.^92^ Hence, it can be speculated that this model will generate a higher amount and resemblance of AD features and can be synergistic compared to individual STZ or APP/PS1 models. Moreover, the characteristics of sporadic and familial AD will probably be overlapped in this newly created animal model. The STZ will be introduced intracerebroventricularly in the same way described previously for mice already having mutated genes in APP and presenilin 1.

### 10.2 Fructose-Induced Model

Like galactose, fructose is a monosaccharide and structural isomer of glucose. It can interfere with the metabolic process by creating insulin resistance and cause imapairment in glucose metabolism. It can stimulate the release of glucocorticoid hormone, thereby controlling food intake. Fructose, if given through diet in an excess amount, has consequences in terms of neuronal death. The brain regions related to food intake and hunger, such as the hypothalamus and hippocampus, can be areas of concern. Finally, it can affect memory impairment and cognitive dysfunction.^93^ Many reports give rise to evidence that AD is primarily a metabolic disease, which can also be characterized by hyperglycemia, hyperinsulinemia, glucose intolerance, and brain insulin resistance.^11,16,17^

### 10.3 Hypertension-Induced AD Model

Increased blood pressure is the excessive pressure exerted on the blood vessels. This is the case when blood gets affected in different body organs, including the brain. Blood usually carries oxygen and other nutrients that are essential for cellular survival. If hypertension persists chronically, it can hinder blood flow to the brain, thereby restricting the brain from obtaining the required nutrients and oxygen. The peripheral hypertensive state can accompany cerebral blood flow hindrance. Mainly, animal hypertension can be induced through drugs or changing lifestyles like minimizing physical movement, high-salt diet, or cholesterol-rich food. Several recent studies have reported hypertension-enhancing cognitive impairment in rats.^94^ Even though the type of dementia incurred through the hypertensive pathway is frequently labeled as vascular dementia, repetitive induction of high blood pressure might also lead to AD-like features.^94^ Again, this is not an AD model but can be combined with the models mentioned earlier to create a more robust model of AD.

## 11 Potential Animal Models in Future Perspective

Even though the models mentioned earlier represent the AD disease model to some extent, there is still no complete resemblance and imitation of the clinical AD manifestations. Therefore, possible alternatives can give rise to more profound AD-related pathological conditions. Some of the strategies can be as follows. Even though the following models have not yet been investigated, these potential options could be investigated in the future for the generation of AD models, which at present have limitations, as mentioned earlier. Furthermore, these potential future models could be the therapeutic targets of anti-AD drugs.

### 11.1 Alloxan-Induced AD Model

Alloxan (160 mg/kg body weight) is a toxic chemical generally having the property of attacking insulin-producing beta cells of the pancreas. Alloxan has already been investigated in the case of the diabetic model; because AD shares some of the features of diabetes, like insulin resistance, alloxan can be examined for inducing brain insulin resistance if administered directly into the brain rather than peripherally. Besides, alloxan also has the potential to generate reactive oxygen species (ROS),^95^ which can further lead to mitochondrial dysfunction, which altogether can be the reason for neurodegeneration. The advantage associated with alloxan is that it is less expensive and readily available as compared to streptozotocin.^95^ Just like STZ, alloxan is also a diabetogenic compound. The STZ-induced AD model is already established. However, the alloxan-induced model has not yet been tried. Therefore, even though alloxan induction causes diabetes, it will still be investigated in the future for a potential AD model.

### 11.2 Acetylcholinesterase-Activated AD Model

Acetylcholinesterase (AchE) is an enzyme ubiquitously located in brain regions and is accountable for the breakdown of acetylcholine, a neurotransmitter involved in synaptic plasticity and memory formation. AchE, its analogs or its activators, the oximes like pralidoxime and obidoxime, can directly be administered in brain regions responsible for cognition and memory regulation. Pralidoxime and obidoxime are generally used for organophosphate poisoning^96^ and treating nerve gas toxicity.

### 11.3 Lesion-Induced Model

Lesions are defined as injuries, damage, or wound infliction in certain areas. Hence, the specific brain regions like the cerebral cortex and hippocampus regulating learning and memory can be made to go through lesion-mediated destruction so that cognitive dysfunction will appear as a symptom of dementia. A bilateral transaction can be made in the hippocampal region to create a learning deficiency. In addition, radiofrequency lesions have also been reported to cause injury.^96^ The neuronal injuries produced by the lesions can finally lead to neurodegeneration. The neurodegeneration in those cognition-controlling areas can be the secondary cause of AD-related pathology. Likewise, the region-specific damages in these brain areas precisely linked to special kinds of memories can be achieved. Therefore, spatial and recognition memory can be observed to be distorted accordingly. However, these super invasive methods can pose severe ethical concerns and the chance of permanent brain damage, and in extreme cases, animal mortality can result. Various advantages and disadvantages of the earlier-mentioned models are tabulated in Table 2.

**Table 2:** Advantages and Disadvantages of the Preclinical Models

models/species	advantages	disadvantages
mammalian transgenic models		
transgenic mice^97^	comparable brain anatomy to humans	crossbreeding/microinjection of transgenic lines expensive and time-consuming
	Aβ plaques and NFT reproducible	strain difference between transgenic lines
	learning and memory performance assessable using behavioral tests	
	therapeutic benefits through examination of histopathology and behavioral tests	
triple transgenic model^50^	age- and region-dependent plaques and tangles development in the 3XTg-AD mice model similar to human AD	high cost for procurement and maintenance
5XAD model^60^	earlier representation of AD features than other transgenic models	phosphorylated tau pathology is less prevalent than amyloid plaques in this model
PDAPP^98^	model shows Alzheimer-like neuropathology; disease progression similar to human; amyloid burden and memory impairment increase with aging	formation of paired helical filament does not accompany neurodegenerative alterations; no global neuronal loss in the cortex region observed through 18 months of age
mammalian nontransgenic models		
nonhuman primates^99^	APP shares same cellular localization, similar structural, biochemical, and age-related changes to human AD	scarcity of brain specimens from aged primates; cost and maintenance is very expensive compared to rodents
dogs^100,101^	homologous similarity in several APP processing genes, ApoE, and presenilin between dogs and humans	dogs do not form dense neuritic plaques and neurofibrillary tangles
		Aβ plaques do not occur in all aged dogs
rabbit^100^	same Aβ peptide sequence to humans	do not develop AD pathology spontaneously
ICV-STZ induced model^102,103^	behavioral similarities to human AD	involved disease pathophysiology is neuro-inflammation but not accumulation of hyperphosphorylated tau and Aβ
	easy disease induction	strong surgical skills and precision in administration required
	single administration required (quick induction)	
amyloid-induced model^104,105^	allows better control on concentration of Aβ, thus better and precise induction of disease	because induction is sudden, progression of disease is not similar to human AD
	single administration required (quick induction)	strong surgical skills and precision in administration required
colchicine-induced model^106^	symptoms of sporadic AD	intracerebroventricular (ICV) administration of colchicine required
	Alzheimer’s like; time-dependent disease progression observed similar to human subjects	strong surgical skills and precision in administration required
scopolamine or atropine-induced model^107,108^	impairs learning and memory	involves degeneration of cholinergic neurons, with little impact on accumulation of hyperphosphorylated tau and Aβ
	can be given orally as well as ICV	
aluminum chloride-induced model^109^	induces memory and cognitive impairment along with Aβ	disease progression nonidentical to human AD
	induces endoplasmic reticulum (ER) stress and oxidative stress	
	easy availability of AlCl_3_	
aged rat model^110^	natural models of memory deficits and dementia	time-consuming
	noninvasive and without any neurochemical manipulations	probability of getting AD phenotypes may vary
high-fat diet-induced model^107^	gives the correlation between diet and AD	time-consuming model
	mimics some features of AD	
guinea pig^110,111^	close similarity to human Aβ sequence	no typical senile plaques and neurofibrillary tangles in the diseased brain
	higher activity of the β-secretase pathway	time-consuming experimental manipulations, low reproduction kinetics
		unavailability of good behavioral study tools
nonmammalian models		
zebrafish^112^	simulates the pathology of Alzheimer’s disease (AD) and tauopathy	amyloid-beta protein shows neurogenesis in the young zebrafish, which can be confusing
	simple nervous system compared to rodents	confirmation of results with higher vertebrate models required
	high-throughput screening	
	ease of genetic manipulation	
*Caenorhabditis elegans* model^113^	admirable molecular genetic model to explore pathways of AD and tauopathies	low translational value
	easy and promising genetic manipulation approaches	multiple pathways cannot be targeted simultaneously
	quick and cheap whole-animal high-throughput screening	far away from mimicking phenotypes of human AD
		no BACE present
Drosophila model^113^	70% of human disease-related genes are conserved in *Drosophila*	a few of the critical features of the pathological signs of AD are not as obviously conserved
	easy and promising genetic manipulation approaches	
	short generation time and short life span	
in vitro models		
chicken embryo primary culture	chick APP gene is identical to humans	no evidence for age-related changes
	helpful to investigate the pathways regulating amyloidosis	
brain slices/brain culture^110,114^	shows the AD mechanism at the molecular and cellular level; controllable environment	no neurological changes, plaques, or neurofibrillary tangles
induced pluripotent stem cells (iPSC)	similarity to human genetic background	no pathological changes
neuroblastoma cell lines^110^	quick and easy to procure	no pathological changes
	set protocols and assay parameters	low translational value
3D human neural cell culture microfluidic model^115^	recapitulate several critical aspects of BBB dysfunction observed in AD patients	

## 12 Conclusion

**Figure 5:**
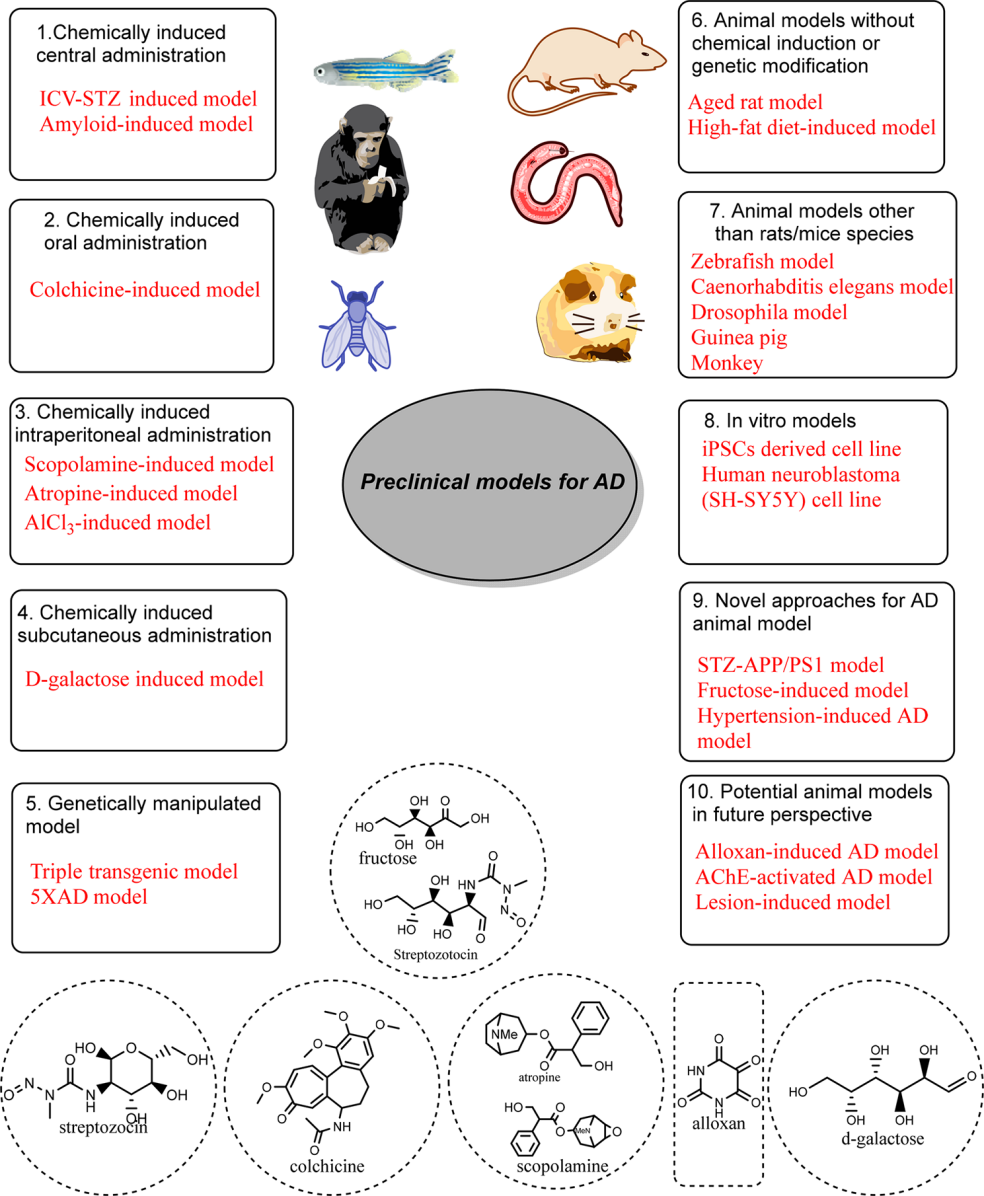
Illustration of various preclinical models for AD.

In a nutshell, all of the past and existing AD models represent pathological features of human AD to some extent but not as a whole. Despite that, the various preclinical AD models with their characteristics of mimicking clinical AD pathology have led to some research opportunities and therapeutic options for clinical AD. Still, newer approaches could be more exploratory for better imitating the disease and reaching a concrete place to understand AD pathology and its subsequent discovery of potential treatments (Figure 5).
